# Association of *preS/S* Mutations with Occult Hepatitis B Virus (HBV) Infection in South Korea: Transmission Potential of Distinct Occult HBV Variants

**DOI:** 10.3390/ijms160613595

**Published:** 2015-06-15

**Authors:** Hong Kim, Bum-Joon Kim

**Affiliations:** Department of Microbiology and Immunology, Liver Research Institute, Cancer Research Institute and SNUMRC, College of Medicine, Seoul National University, Seoul 110-799, Korea; E-Mail: wild0804@snu.ac.kr

**Keywords:** hepatitis B virus infection (HBV), occult infection, South Korea, HBV surface antigen (HBsAg), *preS/S* mutations, genotype C2

## Abstract

Occult hepatitis B virus infection (HBV) is characterized by HBV DNA positivity but HBV surface antigen (HBsAg) negativity. Occult HBV infection is associated with a risk of HBV transmission through blood transfusion, hemodialysis, and liver transplantation. Furthermore, occult HBV infection contributes to the development of cirrhosis and hepatocellular carcinoma. We recently reported the characteristic molecular features of mutations in the *preS/S* regions among Korean individuals with occult infections caused by HBV genotype C2; the variants of *preS* and *S* related to severe liver diseases among chronically infected patients were also responsible for the majority of HBV occult infections. We also reported that HBsAg variants from occult-infected Korean individuals exhibit lower HBsAg secretion capacity but not reduced HBV DNA levels. In addition, these variants exhibit increased ROS-inducing capacity compared with the wild-type strain, linking HBV occult infections to liver cell damage. Taken together, our previous reports suggest the transmission potential of distinct HBV occult infection-related variants in South Korea.

## 1. Introduction

Hepatitis B virus (HBV) infection is a threat to health in many parts of the world, particularly in highly endemic areas, such as the Asia-Pacific region. More than 240 million people are chronic carriers of the virus [1]. HBV infection is associated with a large spectrum of clinical manifestations, ranging from acute or fulminant hepatitis to various forms of chronic infection, including asymptomatic carrier, chronic hepatitis, cirrhosis, and hepatocellular carcinoma (HCC) [2]. The annual number of deaths caused by HBV-related diseases is estimated to be approximately 786,000 worldwide [3]. Because HBV is primarily transmitted vertically from mother to child at birth or during early childhood in highly endemic areas, universal infant vaccination is the most powerful approach to preventing HBV transmission [4,5,6]. HBV infection is endemic in South Korea; based on the Korean National Health and Nutrition Survey of 2011, the prevalence of hepatitis B virus surface antigen (HBsAg) was 3.4% among men and 2.6% among women [7]. HBV vaccination was first introduced to the Korean population in 1983 and dramatically reduced the prevalence of HBsAg-positive chronic carriers from >10% to 3.0% during the approximately thirty-year period that ended in 2011 [7,8]. The immune pressure induced by this successful vaccination likely leads to the generation of diverse mutation patterns that are capable of immune evasion among vaccinated populations. Moreover, the extraordinary prevalence of genotype C2, which is more virulent than genotype B [1] in this area [9], contributes to the distribution of characteristic HBV mutation patterns related to the progression of liver disease. We have identified several HBV variants related to the progression of liver disease, particularly HCC, among Korean patients [9,10,11,12,13,14,15,16,17,18,19,20,21,22,23,24].

## 2. Occult Hepatitis B Virus Infection

Occult HBV infection is defined as the persistence of HBV DNA (primarily in the liver tissue or, in some cases, in the serum) in HBsAg-negative individuals [25,26,27,28]. HBV infection is generally diagnosed when the circulating HBsAg is detected serologically. However, recent progress in molecular-based technologies, such as PCR-based methods, has facilitated the identification of HBV infection in HBsAg-negative individuals with or without circulating antibodies to HBsAg and/or the hepatitis B core antigen (anti-HBc) [29,30,31]. Several mechanisms have been suggested to explain occult infection, including mutations in HBV genes and particularly HBsAg escape mutations, which cannot be detected by commercial HBsAg assays; host mechanisms that lead to strong suppression of HBV replication and transcription; host immune surveillance; co-infection by other viruses, such as hepatitis C virus (HCV); and epigenetic mechanisms [32]. Occult HBV infection can be transmitted via blood transfusion and organ transplantation, including liver transplantation. Furthermore, the development of immune-suppressive conditions could lead to the reactivation of occult HBV infection and the progression of acute or fulminant hepatitis. A large body of evidence indicates that occult HBV infection may be significantly associated with severe forms of liver disease, such as cirrhosis and HCC [33,34]. Furthermore, in HCV-infected patients, occult HBV infection can worsen the course of HCV infection [31,35,36,37].

## 3. Occult HBV Infections in Korean Subjects

The reported prevalence of occult HBV infection among the general population in Guangdong, Hainan, and Long An country, China, is 2.0% (6/294), 3.4% (68/1995) and 11.5% (6/52), respectively [38,39]. In Taiwan, the prevalence of occult HBV infection is 7.5% (8/107) in healthy adults, 4% (9/206) in blood donors, and 10.9% (5/46) in hepatitis B-vaccinated children [40,41,42]. The reported prevalence of HBV occult infection among blood donors in Asia is 1:570–1:7517 in China [43,44], 1:3248 in Hong Kong [45], 1:894–1:1029 in Taiwan [46,47] and 1:3832 in Thailand [48].

Several studies of HBV occult infection in South Korea have been conducted. However, the reported prevalence of this infection in the Korean population varies widely from 0.7% to 16% [21,49,50,51]. This disparity may be due to differences in methodology, target HBV genomes, and analyzed samples. Notably, a previous report indicated that the prevalence of occult HBV infection is significantly higher among subjects with normal ALT levels in South Korea (31 of 195 subjects, 16%) [43]. HBV DNA could be detected in 7 of 47 subjects (15%), even in sera that were negative for both anti-HBs and IgG anti-HBc. In 18 of 24 nested PCR positive subjects, HBV DNA levels exceeded 10^5^ copies/mL, 10–100 times higher than in Western subjects with occult infection [49].

We previously used a nested PCR protocol to amplify a 1378-bp fragment that included a large hepatitis B surface protein (LHB), including *preS1*, *preS2*, and the *S* region, to demonstrate that the prevalence of HBV occult infections in South Korea was approximately 6.6% (41/624 individuals) [21]. To evaluate the prevalence of HBeAg-positive results among our occult subjects, we performed HBeAg serology tests with sera available from 21 of 41 occult subjects. Despite being HBsAg seronegative, 15 of 21 subjects (71.4%) were HBeAg seropositive (Table 1). In particular, one subject (0705-68) exhibited strong HBeAg seropositivity, even higher than that of the positive control (unpublished observation). Phylogenetic analysis based on sequences of 1125 to 1203 bp demonstrated that all 41 strains associated with occult infections in Korean subjects belonged to genotype C2 [21].

**Table 1:** Prevalence of occult HBV infection from in Korean subjects, and the distribution of serotype and HBeAg serostatus of occult subjects.

No. of HBsAg (−) Subjects	No. of Subjects Positive for Nested PCR			Prevalence
624	41			6.6%
	**No. of Serotype (41 subjects ^a^)**			
	*adr*	*adw*	Untypeable	
	39 (95.2%)	1 (2.4%)	1 (2.4%)	
	**No. of HBeAg (+) Subjects (21 subjects ^b^)**			
	15 (71.4%)			

^a^ All forty-one subjects with occult HBV infections were proved to have genotype C2 infections; ^b^ HBeAg serological analysis was performed for 21 of 41 subjects for whom serum samples were available.

We have reported several types of naturally occurring mutations in the *preS* and *S* regions that are related to clinical severity in Korean patients with chronic infection. Notably, these mutations are also observed in Korean individuals with occult infections, suggesting that chronically infected patients, particularly patients infected with some HBV variants associated with clinical severity, may be the major source of occult HBV infection among HBsAg-negative individuals in South Korea [21]. Herein, we provide a comprehensive review focusing primarily on our previous data on *preS* and *S* variants involved in both disease progression among Korean patients with chronic infection [9,10,11,12,13,14,15,16,17,18,19,20,22,23,24,52] and occult infections in Korean patients [21,53].

## 4. Mutations of the Hepatitis B Virus *preS* Region Related to Occult Infection in South Korea

The envelope of HBV is composed of three forms of the HBsAg, which share 226 amino acids at the C-terminus: the LHB (encoded by the *preS1/S2/S* gene), the middle surface protein (encoded by the *preS2/S* gene), and the HBsAg (encoded by the *S* gene). At least two essential functions have been attributed to the *preS* domain during the viral life cycle: attachment to the hepatocyte membrane and budding of the virus in the endoplasmic reticulum (ER) [54,55]. Several lines of evidence indicate that naturally occurring mutations in the *preS* region correlate with a more progressive form of liver disease [56,57,58]. Mutations, particularly deletions, in the *preS* region may affect the ratio between the small and LHBs, resulting in ER stress associated with the aggravation of liver disease [59,60,61]. Furthermore, the integration of a truncated LHB or middle surface protein into the host chromosome enhances the potential development of HCC by increasing the transactivation capacity [62].

Our previous data indicated that a total of 11 types of mutations in the *preS* region, including 8 types of mutations in *preS1* (*i.e.*, *preS1* deletion, W4P/R, K/Q10R, S17A, P32L, W43L/R, H51P, and I84T) and 3 types of mutation in the *preS2* region (*i.e.*, *preS2* deletion, W3R/Stop and S5A), were detected more frequently in Korean subjects with occult infection than in carriers (Table 2 and [21]). Notably, a high frequency of deletions (22 (53.7%) of 41 occult subjects) in the *preS1* (15 subjects) or *preS2* regions (7 subjects) was observed in Korean subjects with occult infections. Interestingly, no subjects had simultaneous deletions in both the *preS1* and *preS2* regions, suggesting that the simultaneous generation of both deletions in a strain may have a lethal effect on the viral life cycle. Of the 2 types of *preS* deletions, most *preS1* deletions (80%, 12/15 subjects) were deletions involving the *preS1* start codon, leading to an 11-amino acid truncation of LHB, as observed in genotype D [21]. This deletion was first reported as a mutation associated with liver disease progression among Korean patients with chronic infection by our group [15]. Furthermore, we recently demonstrated that chronically infected Korean patients with this type of deletion had a significantly higher HBeAg-positive seroprevalence and higher HBV DNA levels than subjects with wild-type HBV [63]. This finding suggests that deletions involving the *preS1* start codon may contribute significantly to disease progression among Korean subjects with chronic HBV infection by extending the duration of HBeAg-seropositive status and increasing HBV replication, which are characteristic traits of highly infective HBV. Thus, together with their high prevalence among Korean subjects with occult infections, the potentially high infectivity of these variants may also contribute to the transmission of occult HBV infection in the Korean population. Notably, one occult-infected subject (0705-68) who exhibited a strongly positive HBeAg serostatus higher than that of the positive control also exhibited a deletion involving the *preS1* start codon (unpublished observations), strongly supporting the above hypothesis.

**Table 2:** *PreS/S* mutations related to occult infections in South Korea.

Regions	Types	Polymerase	Occult (*n* = 41)	Carriers (*n* = 40)	*p*-Value	Clinical Relevance
***preS1***	Hepatocyte receptor binding region	-	29 (70.7%)	7 (17.5%)	<0.001	[58]
	Start codon deletion	spacer6-12 Deletion	11 (26.8%)	3 (7.5%)	<0.05	[14,46]
	W4P/R	spacerL9D	7 (17.1%)	2 (5.0%)	0.087	[21,23]
	K/Q10R	-	6 (14.6%)	0 (0%)	<0.05	-
	S17A	spacerF22C	5 (12.2%)	0 (0%)	<0.05	-
	P32L	-	5 (12.2%)	0 (0%)	<0.05	-
	W43L/R	spacerL48S	6 (14.6%)	0 (0%)	<0.05	-
	H51P	-	5 (12.2%)	1 (2.5%)	0.096	-
	I84T	-	7 (17.1%)	0 (0%)	<0.01	-
***preS2***	Deletion	spacer132-147 Deletion	7 (17.1%)	1 (2.5%)	<0.05	[17,44,47]
	M1L/T/V	-	4 (9.8%)	1 (2.5%)	0.175	-
	W3R/*	-	6 (14.6%)	0 (0%)	<0.05	-
	S5A	-	5 (12.2%)	0 (0%)	<0.05	-
***S***	“a” determinant	-	31 (75.6%)	5 (12.5%)	<0.001	-
	I/T126N/S	-	11 (26.8%)	3 (7.5%)	<0.05	-
	W182L/*	-	15	0 (0%)	<0.001	[19]

The previous mutation analysis in Chinese patients with occult infection by HBV genotype C revealed deletions involving the *preS1* start codon in 2 of 8 patients (25.0%), suggesting that this type of deletion may be common in occult infections in endemic areas of genotype C infection and is not restricted to South Korea [64].

Our previous molecular epidemiology study using *Mbo*-II PRA (PCR Restriction Analysis) demonstrated that the *preS2* deletion was significantly associated with severe forms of liver disease among chronically infected Korean patients [18]. Furthermore, several lines of evidence suggest that the *preS2* deletion correlates with a more progressive form of liver disease by affecting the ratio between HBsAg and LHB, resulting in ER stress associated with the aggravation of liver disease. *PreS2* deletions may also contribute to occult infection by shortening the distance between the S promoter and the transcription initiation site of the *S* mRNA, leading to an altered expression ratio of LHBs to *S* Ag. This altered ratio can also result in HCC via the up-regulation of the ER stress pathway and increased transactivation capacity [58,61]. This finding suggests that overexpression of LHBs among HBV variants with *preS2* deletions may contribute to HBV occult infections by interrupting virion formation. Thus, these results also provide a novel link between occult infection and liver disease progression in genotype C2 infections with *preS2* deletions, particularly among chronically infected Korean patients.

In Chinese patients with genotype C occult infections, no type of *preS2* deletion was detected. Instead, substitutions abolishing the *preS2* start codon and introducing a stop signal at the 3rd codon of *preS2* (W3*), which were also observed in Korean subjects, were detected in 2 patients (25.0%, 2/8) and 1 patient (12.5%, 1/8), respectively [64]. In addition to the 2 types of *preS* deletion (*i.e.*, *preS1* start codon deletion and *preS2* deletion), the W4P/R mutation in the *preS1* region was identified more frequently in occult subjects than in chronic carriers (occult (7/41 subjects) *vs.* carrier (2/40 carriers); *p* = 0.087); however, this difference was not statistically significant (Table 2). Our recent real-time PCR-based molecular epidemiology study reported a novel *preS1* substitution (W4P/R) that was significantly related to severe liver disease among Korean patients who were chronically infected with genotype C (HCC and liver cirrhosis (12.4%, 19/153 patients) *vs.* chronic hepatitis and carrier (1.1%, 1/94 patients), *p* < 0.001). This mutation changes tryptophan to proline or arginine in the 4th codon from the *preS1* start site. Notably, all W4P/R mutations (20 patients) were detected in male patients, suggesting that the W4P/R mutation occurs predominantly in males [18]. Furthermore, our recent study using stable cell lines and a nude mouse xenograft model demonstrated that LHBs containing the W4P mutation could potentiate tumorigenicity and induce gender disparity in an IL-6-dependent manner. Collectively, the results of our study led us to conclude that W4P/R is related to disease severity in male patients chronically infected with genotype C2 and may contribute to occult infections in South Korea [24].

Of the 8 types of mutations in *preS1* related to occult infections, three (*i.e.*, S17A, P32L and W43L/R) are located in the hepatocyte binding site in *preS1*. Significant differences in the prevalence of mutations in the putative hepatocyte receptor region (17th to 43rd codon) were observed between occult subjects and carriers (occult subjects (70.7%, 29/41 subjects) *vs.* carrier (17.6%, 7/40 subjects); *p* < 0.001), suggesting that defects in hepatocyte binding may be related to occult infection in South Korea [65] (Table 2).

## 5. Mutations in the Hepatitis B Virus *S* Region Related to Occult Infection in South Korea

The HBsAg contains a dominant neutralizing epitope termed the “a” determinant in the major hydrophilic region (MHR) of the *S* gene that spans amino acid positions 100–160. The “a” determinant is widely believed to be located between amino acids 124 and 147 of HBsAg, and mutations in this region are associated with the generation of vaccine escape variants and persistent infection [66,67,68,69]. Our previous report revealed two distinct epidemiological traits related to the prevalence of HBsAg variants among chronically infected Korean patients, including an unexpectedly higher prevalence of naturally occurring MHR variants (47/101, 46.5%) and a relatively higher mutation frequency (22/59 (37.3%)), as well as the presence of unique mutation patterns in positions outside the “a” determinant region [11]. These factors could influence virological or clinical aspects of HBV infection among chronically infected Korean patients.

Among the MHR mutations related to vaccine escape, the mutation of glycine to arginine in the 145th codon of HBsAg (*i.e.*, G145R) is detected most frequently worldwide [70,71]. However, the G145R mutation was not observed in any of the 41 Korean patients with occult infections in our previous report. Instead, the 126th codon of HBsAg was most frequently affected by MHR mutations in our samples from patients with occult infection (occult subjects (26.8%, 11/41 subjects) *vs.* carrier (7.5%, 3/40 subjects); *p* = 0.037) (Table 2 and [21]). This finding strongly supports our previous report that MHR mutations of the I/T126N/S type are most prevalent among chronically infected Korean patients with genotype C2 infections [11]. A T126I mutation specific to genotype C was also previously associated with poorer clinical outcomes in patients infected with genotype C, possibly via a change in HBsAg antigenicity [72].

**Figure 1:**
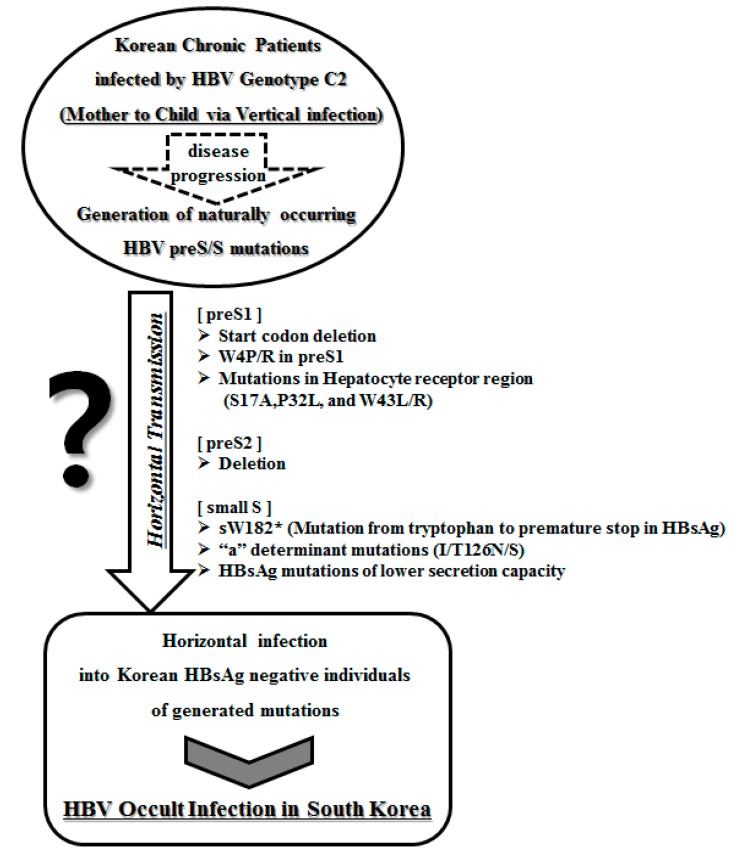
Transmission potential of distinct HBV occult infection-related variants in South Korea.

Notably, including the sW182L and sW182* mutations which have amino acid substitutions from tryptophan (W) to Stop and leucine (L) at the 182th codon of HBsAg, respectively, the 182th codon is the codon most frequently affected in mutations of HBsAg in occult-infected subjects (occult subjects (36.6%, 15/41 subjects) *vs.* carrier (0%, 0/40 subjects); *p* < 0.001) (Table 2). In particular, sW182L was distinctly present in only patients with occult infection and was not observed in chronically infected Korean patients [21]. We previously introduced a novel mutation type outside the MHR regions (*i.e.*, sW182*) that resulted in a premature stop at codon 182 in the HBsAg gene of genotype C2 [20]. Our molecular epidemiology study employing multi-probe real-time PCR demonstrated that the prevalence of this mutation was significantly higher in patients with progressive forms of the disease (*i.e.*, HCC and liver cirrhosis) than in patients with less severe forms of the disease (*i.e.*, chronic hepatitis and carrier) (31.8% (56/176 patients) *vs.* 17.2% (17/99 patients); *p* = 0.010). Furthermore, an *in vitro* study employing stable cell lines expressing HBsAg containing sW182* also strongly supported the relationship of this mutation with HCC [20]. Interestingly, the predominance of sW182* in Korean occult-infected subjects suggests that transfer from chronically infected patients to HBsAg-negative individuals may occur in the Korean population, leading to occult infections (Figure 1). The HBV DNA levels of patients infected with sW182* variants were significantly lower than those infected with wild-type strains, suggesting that these variants may employ mechanisms that reduce the DNA level [20]. The truncated *S* protein may interrupt the formation of normal virions, leading to a loss of infectivity and reduced DNA levels. Consistent with this hypothesis, a study that employed full HBV genomic DNA harboring the sW182* mutation demonstrated that this variant failed to form normal HBV virions [20]. This finding also provides a likely explanation for the prevalence of this variant in subjects with occult HBV infection and in patients with severe types of liver diseases.

In Chinese patients with genotype C occult infections, the G145R mutation was not detected. In 6 of 8 patients (75%), MHR mutations were detected. Stop codon mutations leading to truncated HBsAg, including the sW182* mutation, were also detected in 3 of 8 patients (37.5%) [64].

A specific type of naturally occurring HBsAg mutation may lead to reduced HBsAg secretion [73]. One of the major mechanisms leading to HBV occult infections is the reduction of circulating HBsAg levels [64,74,75,76]. Consistent with these findings, our recent transient transfection study that employed 10 uncommon HBsAg variants from occult-infected Korean subjects also suggested that a major mechanism of occult infection may involve defects in HBsAg secretion rather than alterations in HBsAg antigenicity. However, most of the variants (9/10 variants) exhibited normal virion secretion capacity that was comparable to or higher than that of wild-type virus [53]. This finding provides novel insight into the intrinsic nature of HBV occult infection, which leads to both HBsAg-seronegative status and horizontal infectivity. Furthermore, most of the variants generated more reactive oxidative species than the wild-type virus, increasing the potential for transmission from chronically ill Korean patients, particularly those with severe liver diseases, to the HBsAg-negative Korean population. Although the HBsAg mutations observed in Korean occult-infected subjects were typically associated with reduced levels of secreted HBsAg in comparison to the wild-type virus, as indicated by ELISA, significant differences in the level of secreted HBsAg were observed between variants with different mutation patterns. In particular, specific mutations outside of the “a” determinant (*i.e.*, sW172R and the sW182* and sW182L mutations in the 182th codon) nearly abrogated the secretion of HBsAg, despite being single-point mutations. This finding suggests that these codons may be very important for HBsAg secretion [53]. Considering our recent report implicating the reduced secretion of HBsAg by an occult infection-related variant in the induction of the ER stress pathway [77], HBsAg secretion deficiency caused by the accumulation of occult infection-related HBsAg mutations may represent a link between occult infection and the progression of liver disease.

How the variants associated with apparent HBsAg positive infection in chronic patients lead to HBsAg seronegative infections in occult infection cases remains to be addressed. However, it is tempting to speculate that a difference in the HBV type mediating transmission between chronic infection and occult infection plays a role. As demonstrated in our previous reports, in chronic infection, infection by wild-type HBV rather than by variants may be predominant. Variants could be generated from wild-type via host immune pressure. Therefore, the coexistence of both wild-type and variant HBV may maintain the HBsAg seropositive phase in chronic infection. However, in occult infection, infection by distinct variants may be predominant. Therefore, exclusive infection by variants in the absence of wild-type HBV may be primarily responsible for the HBsAg-seronegative phase in occult infection.

## 6. Conclusions

In conclusion, our previous reports suggest that a deficiency in the HBsAg secretion capacity induced by specific mutations in the *preS* and *S* regions or defects in virion secretion caused by *preS* deletions may play a pivotal role in occult infections with HBV genotype C2 in the South Korean population. Furthermore, the possible accumulation of HBsAg or HBV virions in the ER in the presence of mutations may represent a link between occult infection and liver disease progression. The association of distinct types of variants in the *preS/S* region, such as *preS1* start codon deletion, *preS2* deletion, W4P/R, and sW182*, with clinical severity among chronically infected Korean patients may suggest that these variants are also responsible for the majority of occult infections in South Korea (Figure 1). The possibility of transmission of these variants between Korean populations via organ transplantation or transfusion from donors with occult infections cannot also be excluded.
